# A new regional cotton growth model based on reference crop evapotranspiration for predicting growth processes

**DOI:** 10.1038/s41598-023-34552-7

**Published:** 2023-05-05

**Authors:** Shudong Lin, Mingjiang Deng, Kai Wei, Quanjiu Wang, Lijun Su

**Affiliations:** 1 State Key Laboratory of Eco-Hydraulics in Northwest Arid Region, Xi’an University of Technology, Xi’an, 710048 China; 2 Xinjiang Irtysh River Basin Development and Construction Administrative Bureau, Urumqi, The Xinjiang Uygur Autonomous Region, Ürümqi, 830000 China

**Keywords:** Climate sciences, Environmental sciences

## Abstract

Meteorological conditions and irrigation amounts are key factors that affect crop growth processes. Typically, crop growth and development are modeled as a function of time or growing degree days (*GDD*). Although the most important component of *GDD* is temperature, it can vary significantly year to year while also gradually shifting due to climate changes. However, cotton is highly sensitive to various meteorological factors, and reference crop evapotranspiration (*ET*_*O*_) integrates the primary meteorological factors responsible for global dryland extension and aridity changes. This paper constructs a cotton growth model using *ET*_*O*_, which improves the accuracy of crop growth simulation. Two cotton growth models based on the logistic model established using *GDD* or *ET*_*O*_ as independent factors are evaluated in this paper. Additionally, this paper examines mathematical models that relate irrigation amount and irrigation water utilization efficiency (*IWUE*) to the maximum leaf area index (*LAI*_*max*_) and cotton yield, revealing some key findings. First, the model using cumulative reference crop evapotranspiration (*CET*_*O*_) as the independent variable is more accurate than the one using cumulative growing degree days. To better reflect the effects of meteorological conditions on cotton growth, this paper recommends using *CET*_*O*_ as the independent variable to establish cotton growth models. Secondly, the maximum cotton yield is 7171.7 kg/ha when *LAI*_*max*_ is 6.043 cm^2^/cm^2^, the corresponding required irrigation amount is 518.793 mm, and *IWUE* is 21.153 kg/(ha·mm). Future studies should consider multiple associated meteorological factors and use *ET*_*O*_ crop growth models to simulate and predict crop growth and yield.

## Introduction

With rapid population growth in recent decades, the demand for grain and cash crops has been constantly increasing. To improve crop yield and quality, it is necessary to accurately describe crop growth processes and better control the nutritional factors required for crop growth. A precise understanding of the complex interactions between crops and their surrounding environments is important to predict how environmental conditions affect crop growth processes. Towards this goal, crop growth models have been developed to simulate crop growth and development using complex mathematical functions and modelling techniques^1^. These models can quantitatively and dynamically describe crop growth development and yield formation processes^2^, which is useful for assessing the impact of drought on future crop yields. Additionally, crop growth models can provide detailed estimations of crop status, including phenological status, leaf area index (*LAI*), and yield of specific crop types^3^. Furthermore, these models can predict crop yields as a function of soil conditions, weather, and management practices^4^. Therefore, crop growth models have become important tools for quantitatively evaluating the relationships among soil, weather, and vegetation, and for facilitating the timely regulation of crop growth, which has attracted widespread attention.

Crop growth models have simple forms and are convenient to use^4^, and can be employed to simulate crop growth processes to guide agricultural production. Some models have been adapted to crop breeding to simulate the effects of changes in morphological and physiological characteristics of crops, which helps to identify optimal phenotypes in different environments^5^. In addition, some models have been used to simulate crop growth processes, including the Gompertz^6^, Richards^7^, and logistic^8,9^ models. Pronk^10^ established a dynamic model of dry matter accumulation (*D*) and LAI based on the relationship between wheat and radiation-use efficiency, while Villegas^11^ used the logistic equation to establish a model of *D* of durum wheat based on the crop growth characteristics in a Mediterranean climate. Wang^12^ took the growing degree days (*GDD*) as the independent variable and simulated the dry matter accumulation process in grape fields using logistic, Richards, and Hoerl models. An evaluation of the model results showed that the simulated values using the logistic model were closest to the measured values, indicating that the logistic model was the most accurate.

Crop growth is influenced by many factors, including soil properties, meteorological conditions, nutritional status, and crop species^13^. While soil properties, nutritional status, and crop species can be easily adjusted, meteorological conditions cannot be controlled. They significantly impact agricultural production and show high spatial variability at regional and global scales. Numerous studies have explored the effects of climate variability on crop growth, development, and yield^14,15^. Generally, growth days, such as days after sowing, are used as the independent variable when establishing crop growth models^16^. However, because of differences in meteorological conditions, crop seeding time varies among regions. Furthermore, crop growth is strongly influenced by temperature^17–19^. Temperature plays a central role in the life cycles of crops, affecting growth, development, and yield^20^. Nevertheless, temperature is a highly variable factor that exhibits clear diurnal cycles. To investigate how diurnal variation in temperature affects crop growth stages, the concept of *GDD* was proposed as an ecosystem indicator^21^. *GDD* not only measures accumulated heat but also characterizes the crop growth processes and development in a fixed planting area^22^. *GDD* can be used to analyze thermal conditions under certain meteorological conditions, which can determine the optimal sowing date, growth period, and corresponding physiological growth characteristics of crops^21^. Therefore, some scholars have used *GDD* instead of time to establish crop growth models^9,23,24^. Logistic models based on *GDD*, rather than time, better describe the change processes in major crop growth indicators^9,23–26^. However, *GDD* does not directly reflect the impacts of solar radiation, air humidity, and wind speed on crops, nor does it accurately indicate the effects of high temperatures on crop growth.

Reference crop evapotranspiration (*ET*_*O*_), as the name suggests, is the evapotranspiration from a reference surface like well-watered grass. This value, when multiplied by the crop coefficient based on the crop and its growth stage, gives the value of crop evapotranspiration, which could be considered as the total water requirement, as such, is an important indicator of regional climate change and water cycle status^27,28^. *ET*_*O*_ is calculated using the FAO-56 Penman–Monteith equation, which takes into account temperature, solar radiation, humidity, and wind speed^29,30^. The *ET*_*O*_ has been shown to generally provide reasonable results under a variety of meteorology conditions. Compared to *GDD*, which primarily considers temperature, *ET*_*O*_ can more comprehensively characterize changes in meteorology factors. This means that changes in climate variables due to the effects of global warming will be reflected in the *ET*_*O*_, so it is possible to comprehensively study how meteorology conditions affect crop growth processes at regional scales using *ET*_*O*_.

Cotton (*Gossypium hirsutum* L.) production has far-reaching influences on global economics and social development. Cotton is an important oilseed crop^31^ and one of the most important raw materials for natural textile fibers in the world. Recently, numerous scholars have established cotton growth logistic models based on either time or *GDD*. For example, Wang^24^ developed a cotton growth logistic model based on *GDD*, using the Xinjiang region as an example. Gao^32^ described the biomass accumulation process of main stem leaves, boll subtending leaves, capsule wall, and seed cotton weight using the logistic growth model, while Zhang^16^ used the logistic model to simulate the effects of planting densities and nitrogen rates on cotton biomass accumulation. Although many factors, such as the length of the growing season and soil moisture, affect cotton growth, cotton is especially sensitive to meteorological conditions^20^. Cotton production has gradually shifted to arid and semi-arid regions of the world since the 1950s, which are suitable for growing cotton^33,34^. Four main climate features are most important in cotton production: solar radiation, rainfall, humidity, and temperature^35^. Global warming has accelerated cotton growth and cotton phenology, and the cotton growing period has shortened^36^. Therefore, it is necessary to establish a new cotton growth model that comprehensively considers meteorological impacts on regional cotton growth processes. In this article, we develop a new cotton growth model based on *ET*_*O*_ at the regional scale and evaluate its accuracy using experimental data collected from published papers. This new method can provide insights for analyzing regional cotton growth and its influencing parameters.

## Materials and methods

### Data sources

**Figure 1:**
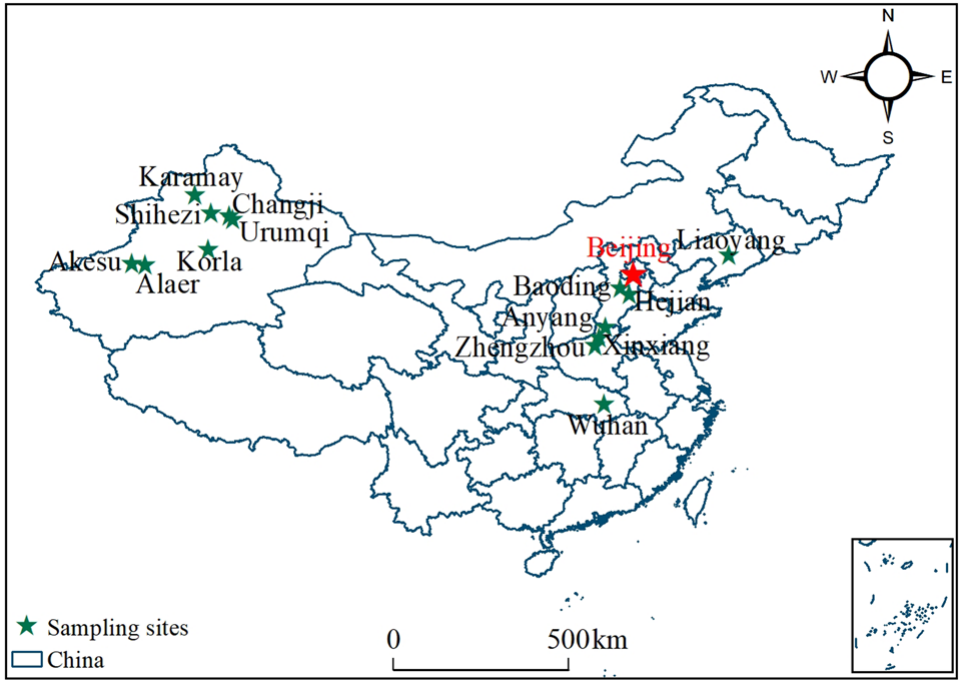
Map of the study area. The stars represent the main data sources. Note: ArcMap 10.5 was employed to generate the map of the study area.

The cotton growth index data used in this study were obtained from previously published studies on cotton growth in China. The portion of the data used for analyzing the characteristics of cotton growth index in Xinjiang came from Wang^24^, and data from other regions are summarized in Table 1. The main cotton planting areas in China are concentrated in Central China, North China, and the Xinjiang region (Fig. 1). In Central China, the climate is characterized by cold winters and hot summers, with frequent drought and flooding. North China has a warm-temperate monsoon climate, with significant seasonal differences in temperature, precipitation, and evaporation. In Xinjiang, the climate is characterized by large temperature ranges throughout the year, low precipitation, and a dry continental climate. The sowing times for different varieties of cotton in different regions range from mid-April to mid-May, with harvest times from late September to late October.

**Table 1:** Sample sizes and data sources by region.

Province	City	Sample size	References	Remarks (variety information)
Hebei	Baoding	6	^37^	Insect-resistant transgenic Bt gene xin cotton 33B
	Hejian	3	^38^	Guoxin cotton 3
He’nan	Zhengzhou	4	^39^	*Bt Cry1A* + *CpTI* and hybrid cotton 72
	Xinxiang	30	^40^	Medium cotton 50
	Anyang	9	^41^	Medium cotton 50; Lu cotton 28; Xin cotton 99B
Liaoning	Liaoyang	8	^42^	Liao cotton 19
Hubei	Wuhan	10	^43,44^	KB02F_1_; Bt insect-resistant cotton GK19

Meteorological data were collected from the National Meteorological Information Center of the China Meteorological Data Service Center (http://data.cma.cn/) and the ERA5 hourly data on single levels from 1979 to present (https://doi.org/10.24381/cds.adbb2d47). The data were used to analyze the effects of meteorological conditions on regional cotton growth. Specifically, we collected data on temperature, solar radiation, humidity, wind speed, and precipitation. To collect cotton growth index data, we used the GetData Graph Digitizer to extract data directly from text or figures in published studies. More than three sets of data samples were selected from most regions, but a few areas had only 1–3 sets of data samples. Table 1 shows the sample sizes and data sources for cotton growth index data from in China, including North China and Central China region.

### Data processing method and error analysis

All data were processed in Microsoft Office Excel (Microsoft Corporation) and MATLAB (MathWorks Inc., Natick, MA, USA) was used for model parameter calculation. ArcMap 10.5 was employed to generate the map of the study area. To address the potential errors or bad numbers that may have arisen during the data digitization process, we took the following methods to minimize their impact on our analysis: i) We carefully reviewed all digitized data points to check for any obvious outliers or inconsistencies, and corrected any that were identified. ii) We compared the digitized data with the original data source and conducted three repetitions of the same digitized data to ensure that there were no significant differences. Correlations were assessed using the *R*^2^, and the accuracy was evaluated through RMSE and RE, as follows:

1 $$ R^{2} = \frac{{\left[ {\sum {\left( {x_{i} - \overline{x} } \right)\left( {y_{i} - \overline{y} } \right)} } \right]^{2} }}{{\sum {\left( {x_{i} - \overline{x} } \right)^{2} \sum {\left( {y_{i} - \overline{y} } \right)^{2} } } }} $$

2 $$ RMSE = \sqrt {\frac{{\sum {\left( {m_{vi} - c_{vi} } \right)^{2} } }}{n}} $$

3 $$ RE = \frac{{\sum {\left( {m_{vi} - c_{vi} } \right)^{2} } }}{{\sum {m_{vi}^{2} } }} $$

where, *x*_*i*_ is the independent variable; *y*_*i*_ is the dependent variable; $\overline{x}$ and $\overline{y}$ represent the average values of *x*_*i*_ and *y*_*i*_, respectively; *m*_*vi*_ is the measured value; and *c*_*vi*_ is the calculated value.

## Mathematical model development and construction

### Cotton growth model based on cumulative growing degree days

Every crop has biologically defined upper and lower temperature limits beyond which its growth and development can be adversely affected. For cotton, the upper and lower temperature limits are 40 ℃ and 10 ℃, respectively, as demonstrated by numerous studies^24,45–47^. Growing degree days (*GDD*) are a measure of heat accumulation used to estimate crop development and growth. In this study, we converted daily *GDD* data to cumulative *GDD* (*CGDD*) using the following equation:

4 $$ CGDD = \sum {(T_{avg} - T_{base} )} $$

where, *CGDD* is the cumulative growing degree days, ℃; *T*_*avg*_ is the mean daily temperature, ℃; and *T*_*base*_ is the minimum daily temperature required for crop activity, ℃. McMaster and Wilhelm proposed a method for calculating *T*_*avg*_^48^:

5 $$ T_{avg} = \left\{ \begin{gathered} \frac{{(T_{x} + T_{n} )}}{2} \, if \, T_{base} < T_{avg} < T_{upper} \hfill \\ T_{base} \quad \quad \, if \, T_{avg} \le T_{base} \hfill \\ T_{upper} \quad \quad \, if \, T_{avg} \ge T_{upper} \hfill \\ \end{gathered} \right. $$

where, *T*_*upper*_ is the maximum temperature at which crop activities continue, ℃; *T*_*x*_ is the highest daily temperature, ℃; and *T*_*n*_ is the lowest daily temperature, ℃.

A logistic model describing plant height (*H*), leaf area index (*LAI*), and dry matter accumulation (*D*) of a crop using *CGDD* as the independent variable was established. Equations to calculate the relative change of indicators of *H*, *LAI*, and *D* are used to minimize the differences in cotton growth indicators caused by irrigation methods or soil conditions in different regions. The relative cotton growth index and *CGDD* are obtained using these corrected indicators. The logistic models between the relative *CGDD* (*R*_*CGDD*_) and relative *H*, relative *LAI*, and relative *D*, are expressed as:

6 $$ R_{H} = \frac{H}{{H_{\max } }} = \frac{1}{{1 + e^{{a_{1} + b_{1} R_{CGDD} }} }} $$

7 $$ R_{LAI} = \frac{LAI}{{LAI_{\max } }} = \frac{1}{{1 + e^{{a_{2} + b_{2} R_{CGDD} + c_{2} R_{CGDD}^{2} }} }} $$

8 $$ R_{D} = \frac{D}{{D_{\max } }} = \frac{1}{{1 + e^{{a_{3} + b_{3} R_{CGDD} }} }} $$

where, *R*_*H*_ is the relative plant height; *H* is the plant height, cm; *H*_*max*_ is the theoretical maximum *H*, cm; *R*_*LAI*_ is relative leaf area index; *LAI* is leaf area index, cm^2^/cm^2^; *LAI*_*max*_ is the theoretical maximum *LAI*, cm^2^/cm^2^; *R*_*D*_ is relative dry matter accumulation; *D* is dry matter accumulation, g/plant; *D*_*max*_ is the theoretical maximum *D*, g/plant;* R*_*GDD*_ is the relative *CGDD*; and *a*_1_, *a*_2_, *a*_3_, *b*_1_, *b*_2_, *b*_3_, and *c*_2_ are model fitting parameters. The maximum measured value of each index in a field experiment may not actually reach the maximum value, so we increased the values using multiplication factors (for example, *H*_*max*_ was multiplied by an incremental factor of between 1.01 and 1.05) to reach the theoretical maximum value^8,24^.

### Cotton growth model based on cumulative reference crop evapotranspiration

The crop growth model based on *GDD* only accounts for the effect of temperature on crop growth, but cotton growth is influenced by other meteorological factors as well. Therefore, using *GDD* alone is insufficient to accurately model cotton growth. Instead, we used reference evapotranspiration (*ET*_*O*_), which considers various meteorological conditions such as temperature, solar radiation, water vapor pressure, and wind speed. The Penman–Monteith method is a widely accepted method for calculating *ET*_*O*_, as it incorporates both physiological and meteorological parameters. We used the FAO-56 Penman–Monteith method, which is a standard and reliable approach used worldwide. To account for cumulative evapotranspiration over time, we calculated cumulative reference crop evapotranspiration (*CET*_*O*_) using daily *ET*_*O*_ data and a specific equation.

9 $$ CET_{O} = \sum {ET_{O} } = \sum {\frac{{0.408\Delta (R_{n} - G) + \gamma \left( {\frac{900}{{T + 273}}} \right)u_{2} (e_{s} - e_{a} )}}{{\Delta + \gamma (1 + 0.34u_{2} )}}} $$

where, *R*_*n*_ is the net radiation, MJ/m^2^/d; *G* is the soil heat flux, MJ/m^2^/d; $\gamma$ is the psychrometric constant, kPa/°C; Δ represents the slope of the saturation vapor pressure versus temperature curve, kPa/°C; *e*_*s*_-*e*_*a*_ is the vapor pressure deficit, kPa; *T* is the average atmospheric temperature, °C; and *u*_2_ is the wind speed at approximately 2 m above ground level, m/s.

Thus, the logistic model with *CET*_*O*_ as the independent variable is expressed as:

10 $$ R_{H} = \frac{H}{{H_{\max } }} = \frac{1}{{1 + e^{{d_{1} + e_{1} R_{{CET_{o} }} }} }} $$

11 $$ R_{LAI} = \frac{LAI}{{LAI_{\max } }} = \frac{1}{{1 + e^{{d_{2} + e_{2} R_{{CET_{o} }} + f_{2} R_{{CET_{o} }}^{2} }} }} $$

12 $$ R_{D} = \frac{D}{{D_{\max } }} = \frac{1}{{1 + e^{{d_{3} + e_{3} R_{{CET_{o} }} }} }} $$

where, *R*_*CETo*_ is relative *CET*_*O*_; and* d*_1_, *d*_2_, *d*_3_, *e*_1_, *e*_2_, *e*_3_, and *f*_2_ are model fitting parameters.

## Model evaluation

### Growth model of plant height

**Figure 2:**
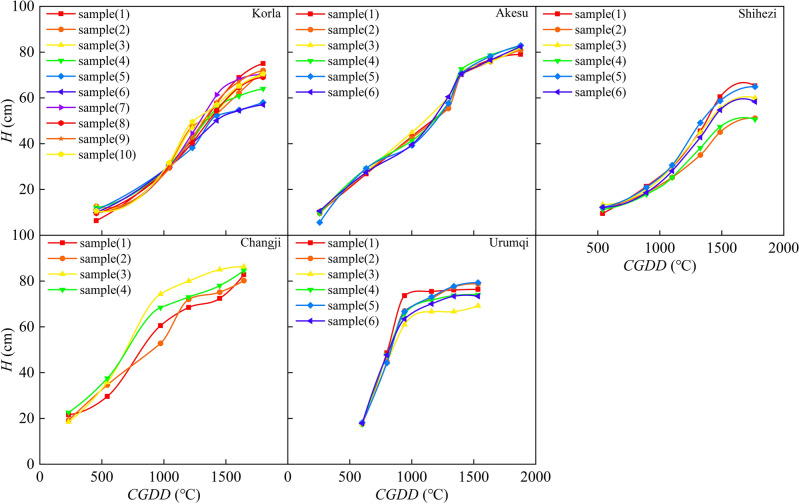
Relationship between cotton plant height and cumulative growing degree days in different regions.

**Figure 3:**
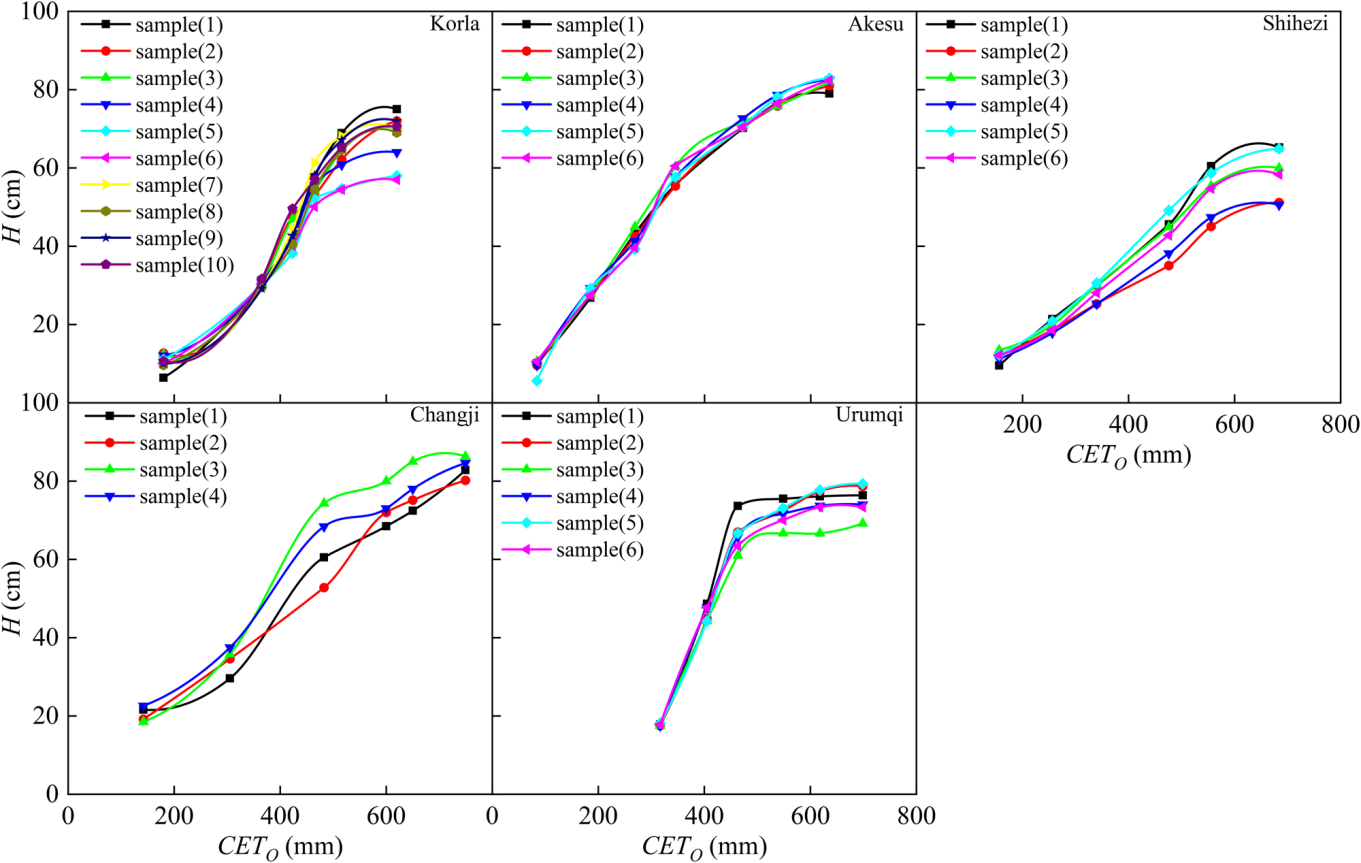
Relationship between cotton plant height and cumulative reference crop evapotranspiration in different regions.

Plant height (*H*) is a useful indicator of crop growth and nutritional status^49^, and it is closely related to *CGDD* and *CET*_*O*_. Several studies have demonstrated that *H* can accurately estimate aboveground biomass and *LAI* of crops^50,51^. We analyzed the relationship between *H* and *CGDD* and *CET*_*O*_, using partial samples from different regions, as shown in Figs. 2 and 3. The growth curve of *H* for both indicators showed a similar "S" shape, indicating slow growth in the early stage, rapid growth in the middle, and slow growth in the later period. This pattern suggests that high temperatures and sufficient light promote rapid growth in the middle stage, whereas low temperatures and reduced sunshine in the later period lead to slower growth. In southern Xinjiang, *H* peaked at approximately 1800 ℃ *CGDD* and 600 mm *CET*_*O*_, while in northern Xinjiang, *H* peaked at around 1600 ℃ *CGDD* and 700 mm *CET*_*O*_. The difference in *CGDD* between southern and northern Xinjiang was mainly due to temperature, with low precipitation and high temperatures in the former and vice versa in the latter. In contrast, *CET*_*O*_ reflects various meteorological conditions, and thus the difference in *CET*_*O*_ between the two regions was smaller. This suggests that *CET*_*O*_ is a more comprehensive indicator for regulating cotton *H*, as it considers multiple meteorological factors.

**Figure 4:**
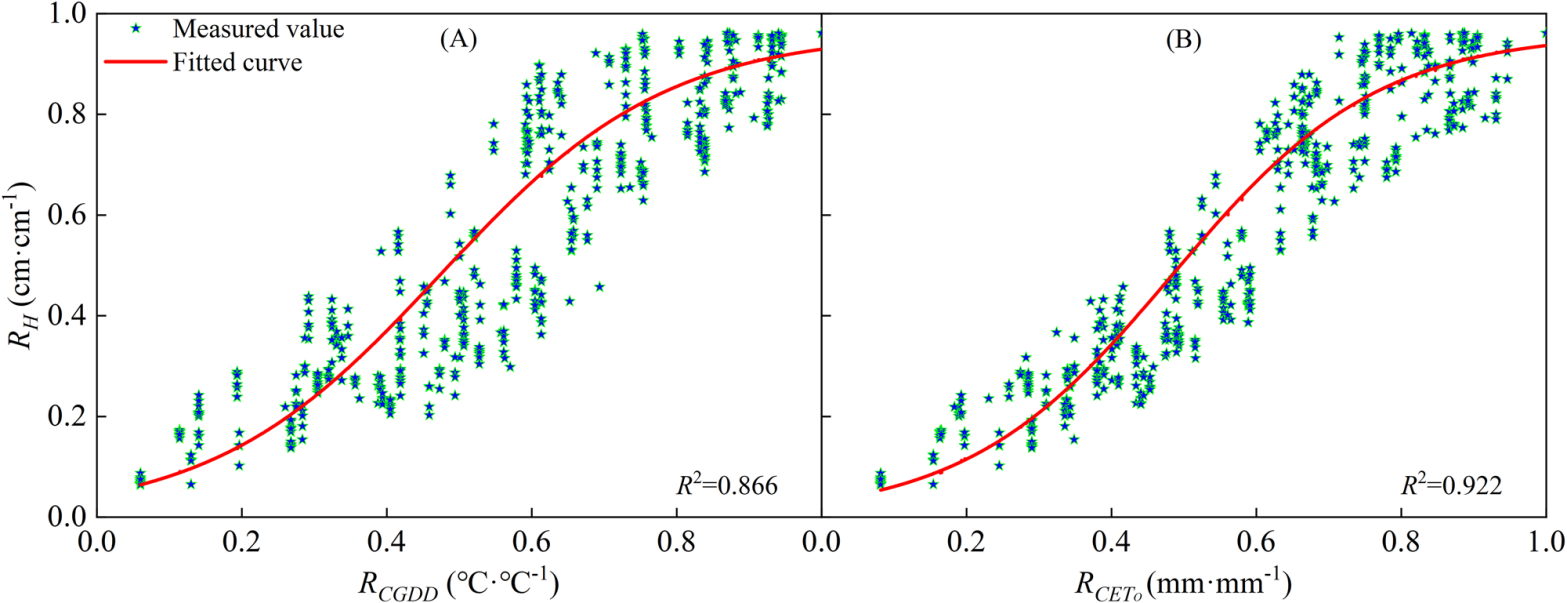
Relationships of relative plant height with relative cumulative growing degree days (**A**) and relative cumulative reference crop evapotranspiration (**B**).

The characteristics of *H* change were similar across all regions, indicating a consistent pattern of growth regulation. To normalize the *H* change characteristics, we established relative logistic models of cotton *R*_*H*_ with *R*_*CGDD*_ and *R*_*CETo*_, using data from different regions. The *R*_*H*_ change curves, along with the fitting results, are presented in Fig. 4 (*P* < 0.05). These models provide a useful framework for analyzing the effect of different meteorological conditions on cotton growth, and can be used to identify the optimal conditions for maximizing *R*_*H*_ and other growth indicators. The *R*_*H*_ model were established as follows:

13 $$ R_{H} = \frac{H}{{H_{\max } }} = \frac{1}{{1 + e^{{2.82 - 5.424R_{CGDD} }} }} $$

14 $$ R_{H} = \frac{H}{{H_{\max } }} = \frac{1}{{1 + e^{{3.212 - 6.091R_{{CET_{o} }} }} }} $$

**Figure 5:**
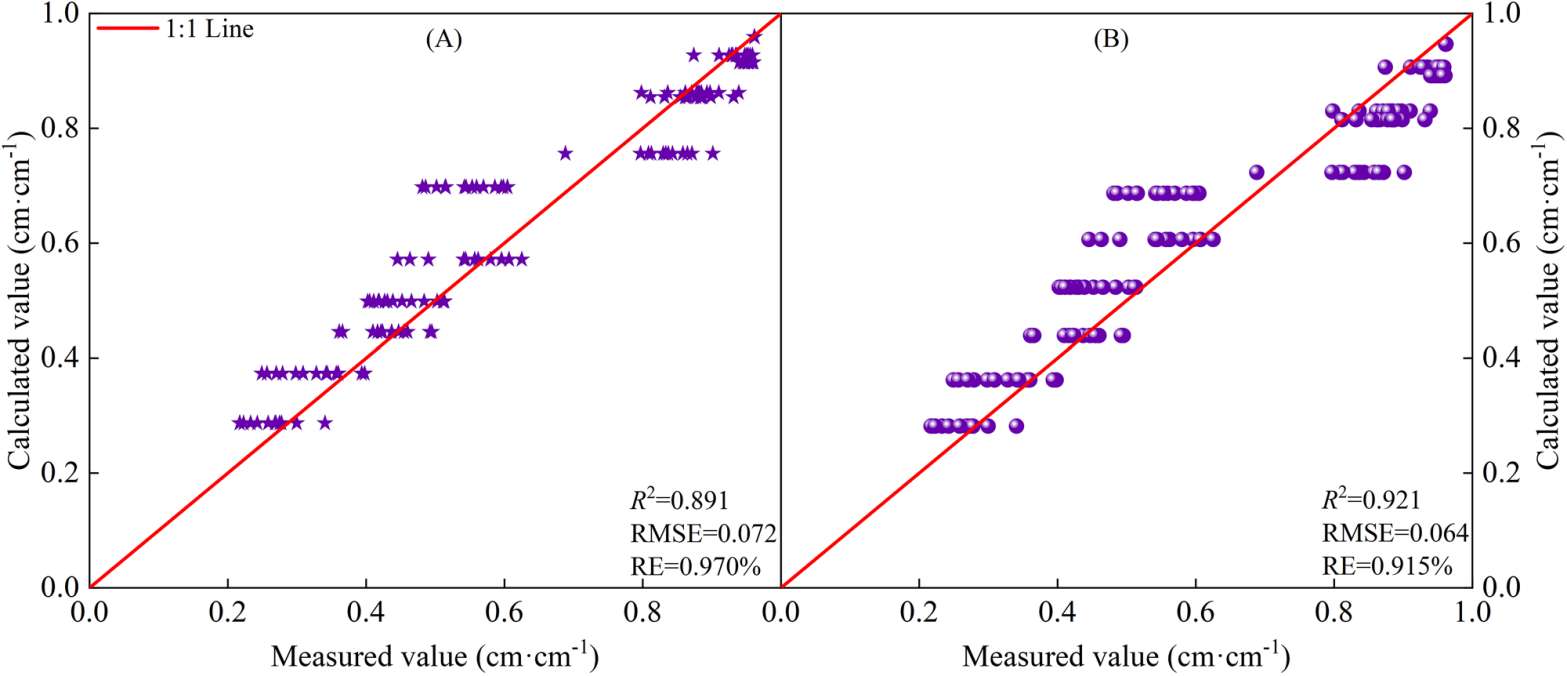
Model validation results of relative plant height and relative cumulative growing degree days (**A**), relative cumulative reference crop evapotranspiration (**B**).

Figure 4 shows the fitting results of *R*_*H*_ of cotton based on *CGDD* and *CET*_*O*_ in different regions, the *R*^2^ ≥ 0.866, indicating that the model had a good fit and high degree of precision. Moreover, it can be seen that the fitting result where *R*_*CETo*_ was the independent variable is better than with *R*_*CGDD*_, and the discrete level of *R*_*CETo*_ was smaller than that of *R*_*CGDD*_. Those experimental data that were not included in the modeling were used to further validate the model (Fig. 5 (A and B); *R*^2^ ≥ 0.891, RMSE ≤ 0.072, RE ≤ 0.970%). There was good agreement between the measured and calculated values of *R*_*H*_. These results clearly showed that the model with *R*_*CETo*_ as the independent variable was more accurate than that with *R*_*CGDD*_.

### Growth model characteristics of cotton leaf area index

**Figure 6:**
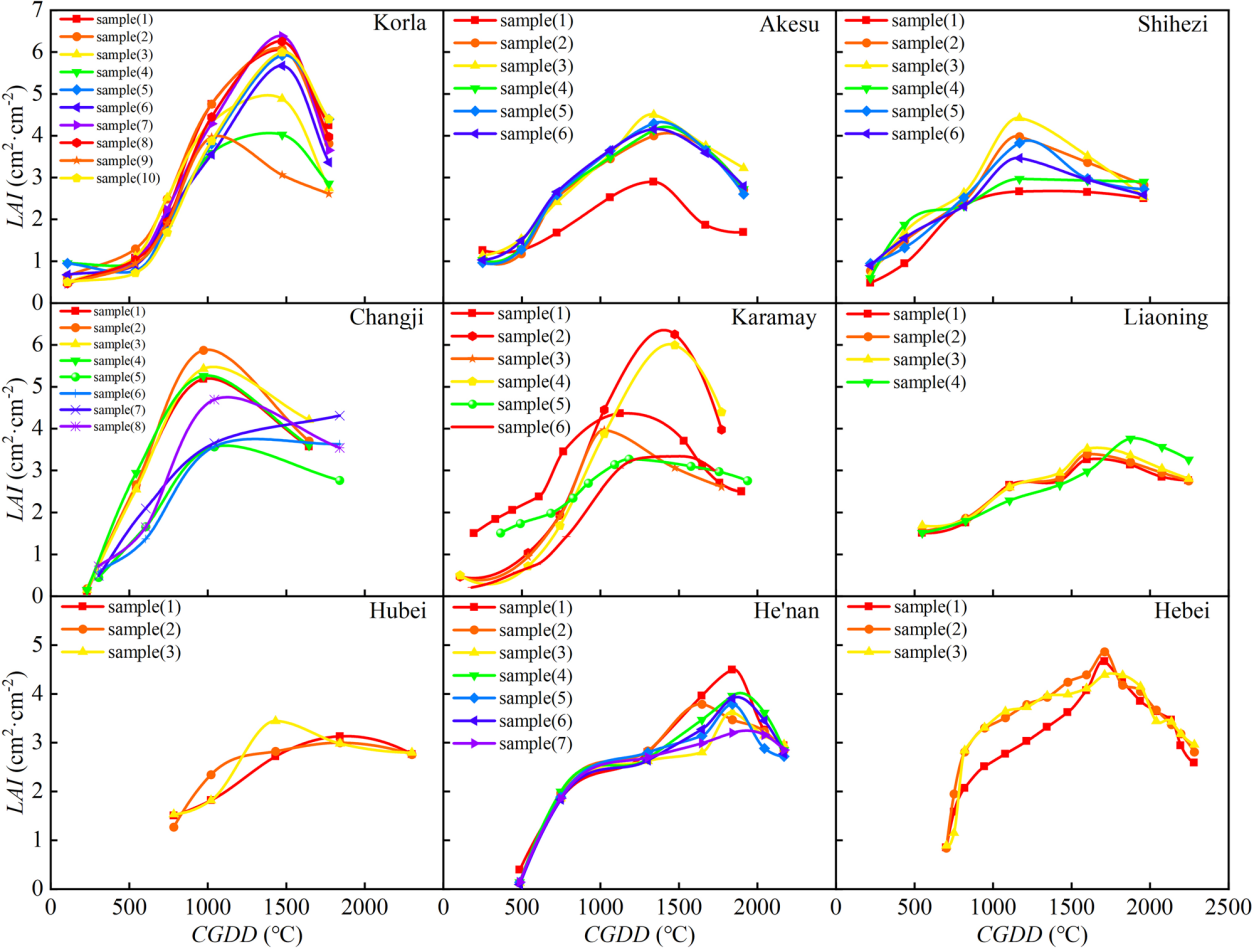
Relationship between cotton leaf area index and cumulative growing degree days in different regions.

**Figure 7:**
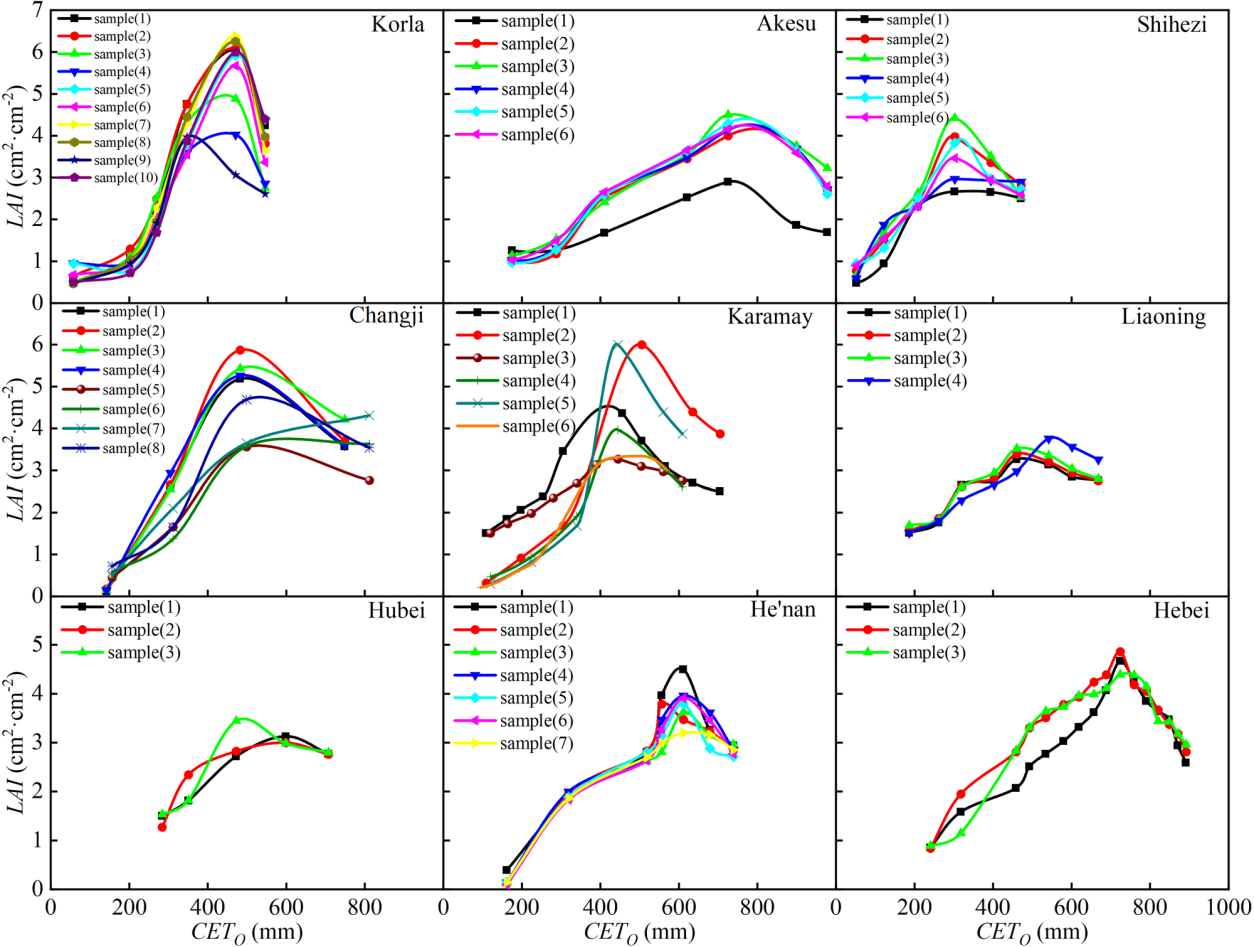
Relationship between cotton leaf area index and cumulative reference crop evapotranspiration in different regions.

As an important quantitative indicator of each growth stage of cotton, *LAI* plays a very important role in assessing growth status. The cotton *LAI* change processes with *CGDD* and *CET*_*O*_ are shown in Figs. 6 and 7, respectively. The *LAI* increased with *CGDD* and *CET*_*O*_ in the early cotton growth stage, but gradually decreased in the late growth period. Photothermal resources are highly important and cotton growth requires sufficient temperature and light. When the *CGDD* was about 1400 ℃, *LAI* reached its maximum value in Xinjiang. When the *CGDD* was about 1600 ℃, *LAI* reached its maximum value in Central China and North China. However, when *CET*_*O*_ was used to describe the *LAI* growth process, the *CET*_*O*_ values when the *LAI* reached its maximum in different regions varied greatly. This was because meteorological conditions varied greatly among regions, and while *CGDD* is only sensitive to temperature, *CET*_*O*_ integrates many meteorological factors, which creates a more realistic predictor of the growth process of *LAI*. This further showed that replacing *CGDD* with *CET*_*O*_ will improve the accuracy of a crop growth model.

**Figure 8:**
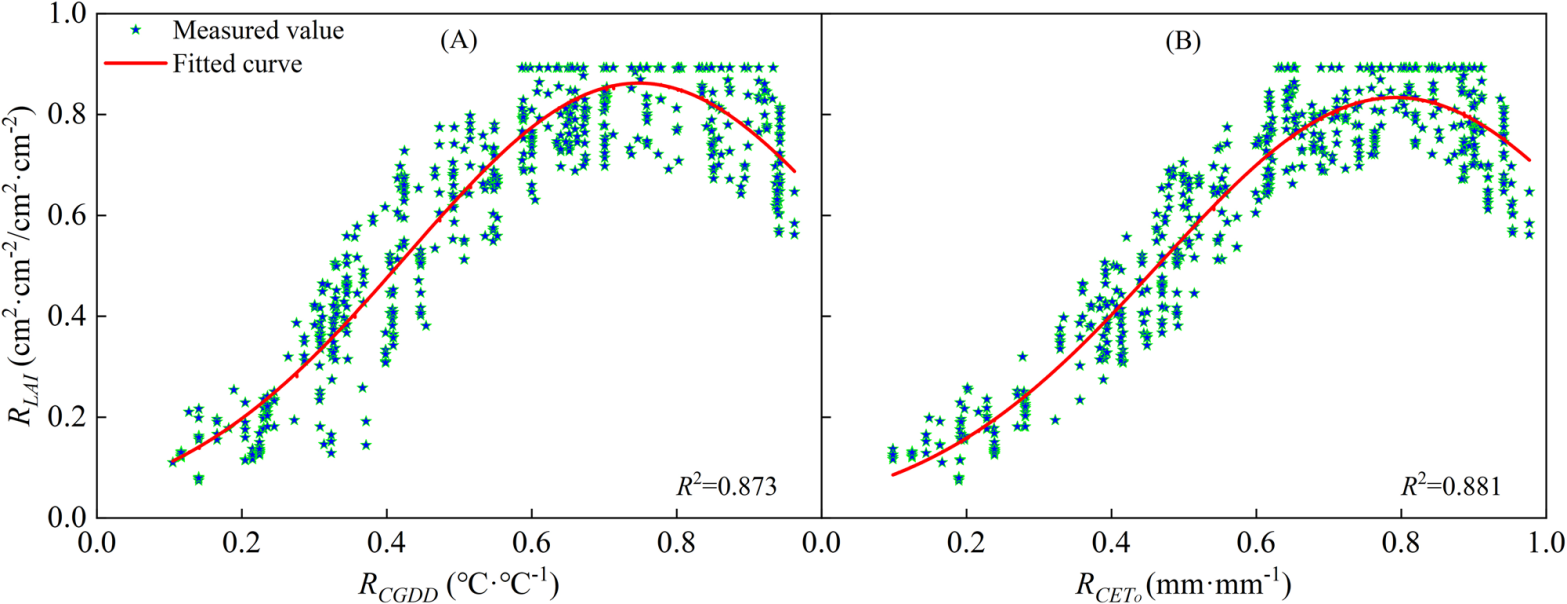
Relationships of relative leaf area index with relative cumulative growing degree days (**A**) and relative cumulative reference crop evapotranspiration (**B**).

It can be seen from Figs. 6 and 7 that there were differences in the *LAI* among different regions due to varying conditions, such as soil fertility, irrigation, and fertilization systems. Despite these differences, the change trends in *LAI* remained consistent throughout the growth period. The change characteristics of the *LAI* were analyzed and compared in different regions to determine the universal change characteristics. Thus, logistic models of cotton *R*_*LAI*_ related to *R*_*GDD*_ and *R*_*CETo*_ were established, as shown in Fig. 8. The curve's rate of change was relatively high in the early stages of cotton growth, indicating that appropriate meteorological conditions can significantly enhance leaf area growth during this period. The *R*_*LAI*_ was fit to the models as shown in Fig. 8 (*P* < 0.05). The *R*_*LAI*_ model was established as follows:

15 $$ R_{LAI} = \frac{LAI}{{LAI_{\max } }} = \frac{1}{{1 + e^{{4.553 - 16.03R_{CGDD} + 10.09R_{CGDD}^{2} }} }} $$

16 $$ R_{LAI} = \frac{LAI}{{LAI_{\max } }} = \frac{1}{{1 + e^{{5.024 - 15.66R_{{CET_{o} }} + 9.5R_{{CET_{o} }}^{2} }} }} $$

**Figure 9:**
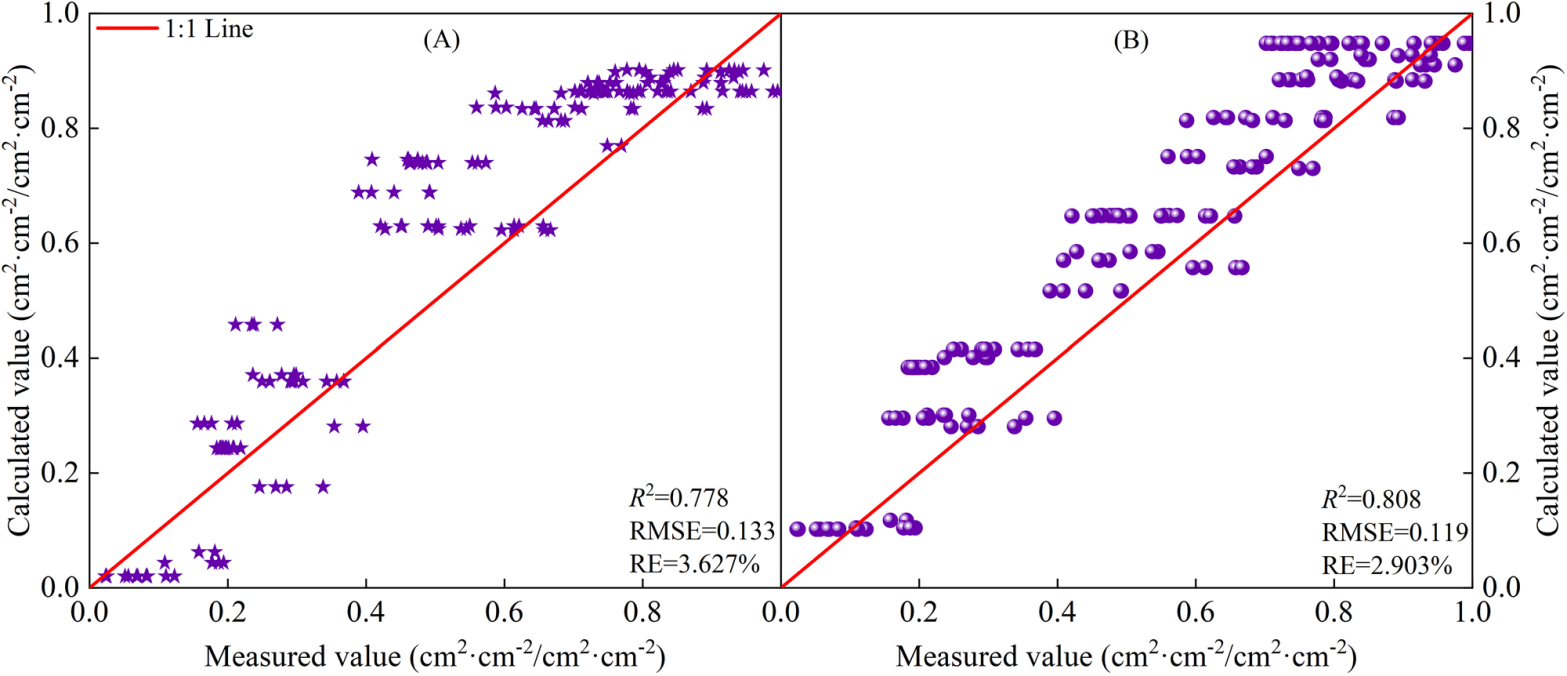
Model validation results of relative leaf area index and relative cumulative growing degree days (**A**), relative cumulative reference crop evapotranspiration (**B**).

The based on *CGDD* and *CET*_*O*_ model fitting and validation results are shown in Figs. 8 and 9, with *R*^2^ ≥ 0.778, RMSE ≤ 0.133, and RE ≤ 3.627%. The fitting effect when using *R*_*CETo*_ as the independent variable was better than when using *R*_*CGDD*_, and the discrete level of *R*_*CETo*_ was smaller than *R*_*CGDD*_. If $\frac{{dR_{LAI} }}{{dR_{CGDD} }} = 0$, $\frac{{dR_{LAI} }}{{dR_{{CET_{O} }} }} = 0$, we know that the *R*_*LAI*_ maximum occurred when *R*_*CGDD*_ and *R*_*CETo*_ were 0.794 and 0.824, respectively. These results showed that *R*_*CGDD*_ and *R*_*CETo*_ were notably different when *R*_*LAI*_ peaked, further indicating that *CET*_*O*_ is a better indicator than *CGDD*.

### Growth model characteristics of cotton dry matter accumulation

**Figure 10:**
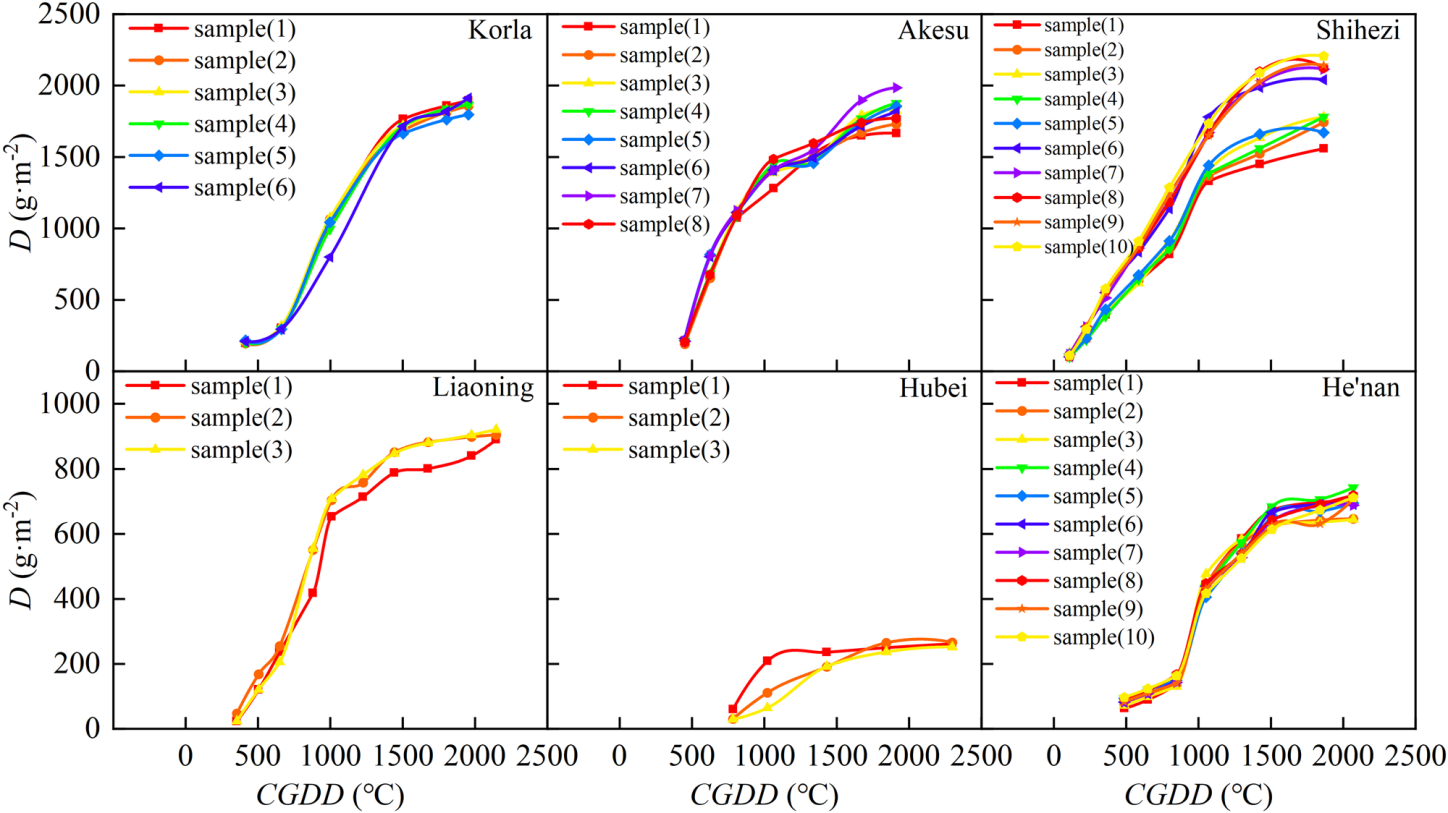
Relationship between cotton dry matter accumulation and cumulative growing degree days in different regions.

**Figure 11:**
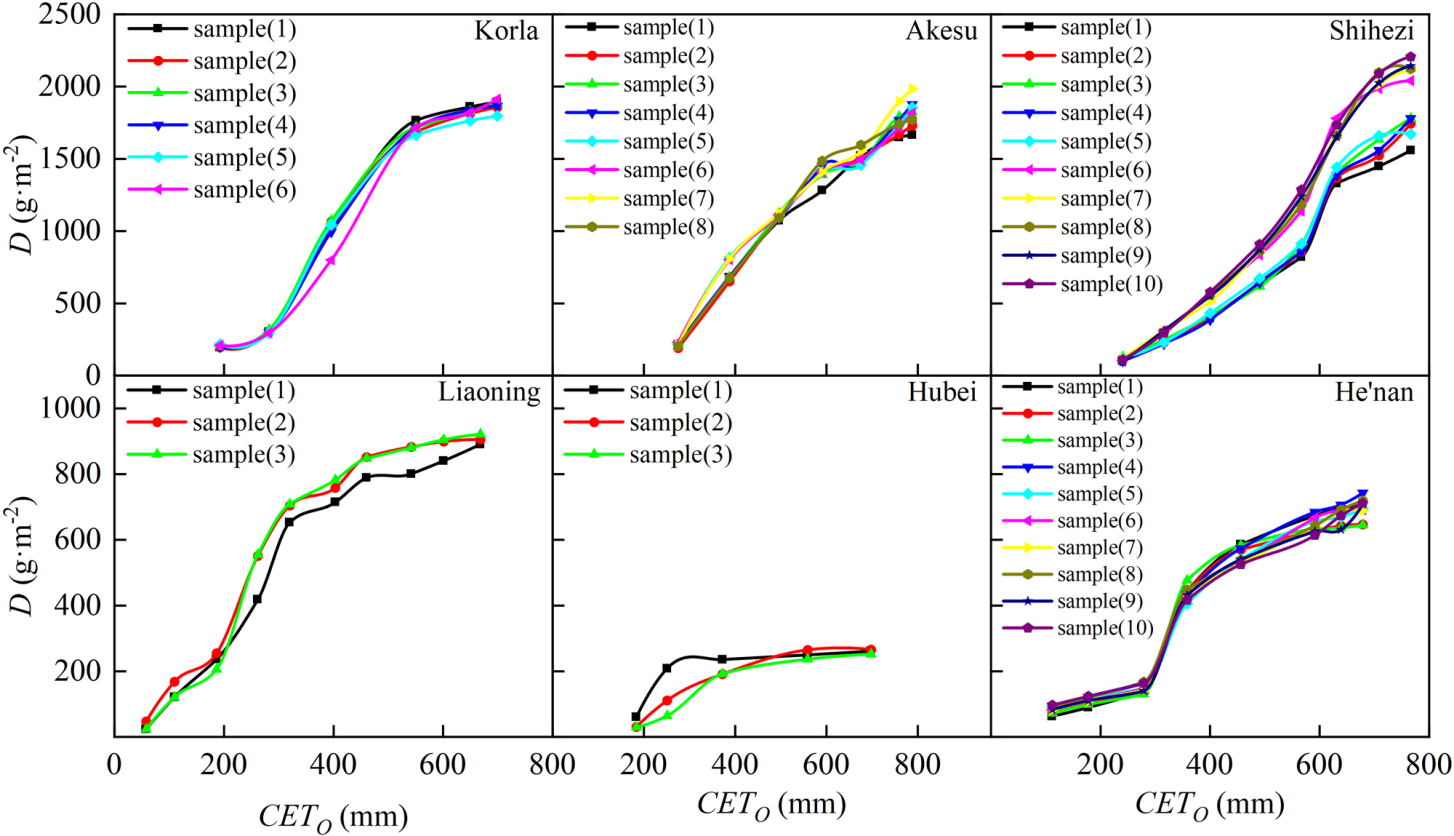
Relationship between cotton dry matter accumulation and cumulative reference crop evapotranspiration in different regions.

Crop yield is based on dry matter production, which is determined by nutrient absorption^52^. Dry matter accumulation is an essential indicator of cotton growth and is crucial for achieving high-quality and high-yielding cotton crops. The trends in the increase and decrease of cotton dry matter were similar among different regions. The rate of change of dry matter accumulation peaked when *CGDD* was between 700 and 1000 °C (Fig. 10) and *CET*_*O*_ was between 200 and 500 mm (Fig. 11), during the budding stage, flowering stage, and boll development stage. During these stages, many leaves grew, and photosynthesis significantly increased due to sufficient temperature and light availability, leading to high cotton yields. Dry matter generally accumulated throughout the growing season, starting slowly during the seedling stage, gradually accelerating during the budding stage, peaking during the flowering to boll setting stages, and then stabilizing. However, due to the arid climate in Xinjiang, with high evaporation and little precipitation, dry matter accumulation peaked when *CGDD* was about 1900 °C and *CET*_*O*_ was about 750 mm. The peak *CGDD* for dry matter accumulation was lower in Xinjiang than in Central China and North China, while the peak *CET*_*O*_ was higher in Xinjiang than in Central China and North China.

**Figure 12:**
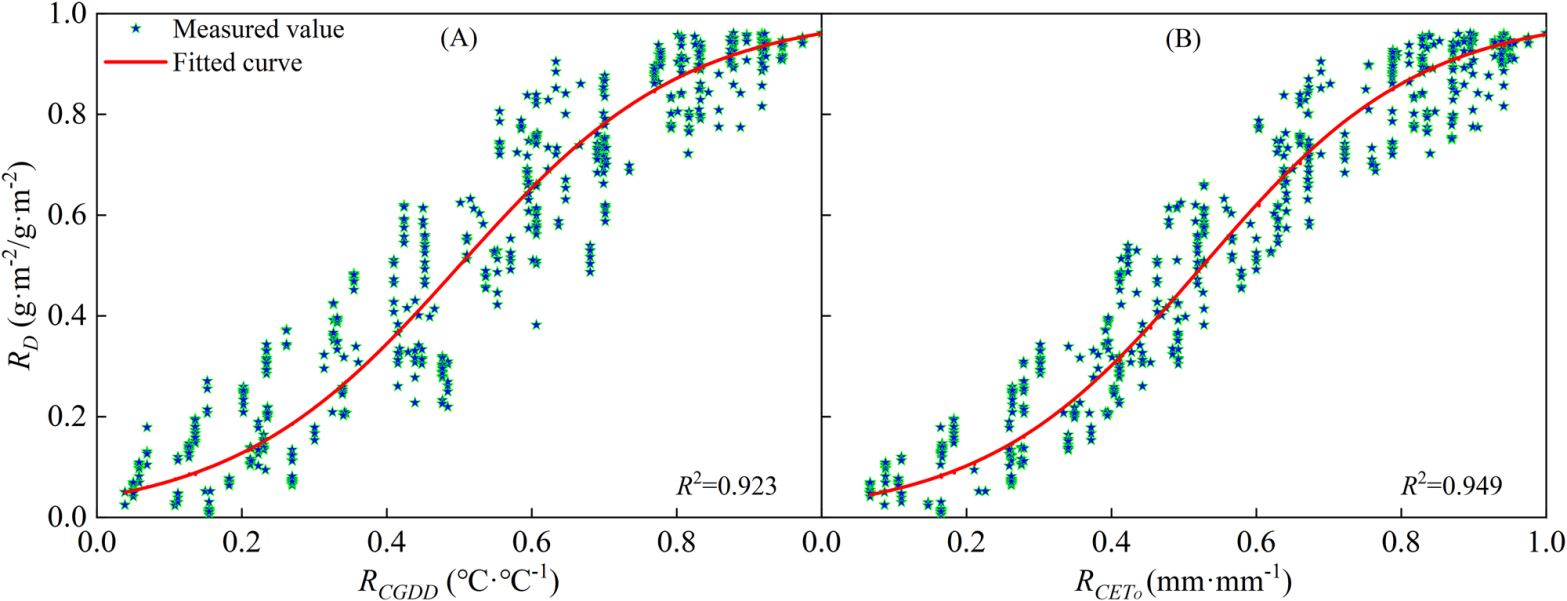
Relationships of relative dry matter accumulation with relative cumulative growing degree days (**A**) and relative cumulative reference crop evapotranspiration (**B**).

The change characteristics of the cotton *D* in separate regions were analyzed and compared to reveal the universal change characteristics of *D.* Thus, the logistic models of the change processes of cotton *R*_*D*_ with *R*_*CGDD*_ and *R*_*CETo*_ were established, as shown in Fig. 12. The fitting result is shown in Fig. 12 (*P* < 0.05), with *R*_*D*_ calculated using the following formulas:

17 $$ R_{D} = \frac{D}{{D_{\max } }} = \frac{1}{{1 + e^{{2.877 - 5.764R_{CGDD} }} }} $$

18 $$ R_{D} = \frac{D}{{D_{\max } }} = \frac{1}{{1 + e^{{3.206 - 6.163R_{{CET_{o} }} }} }} $$

**Figure 13:**
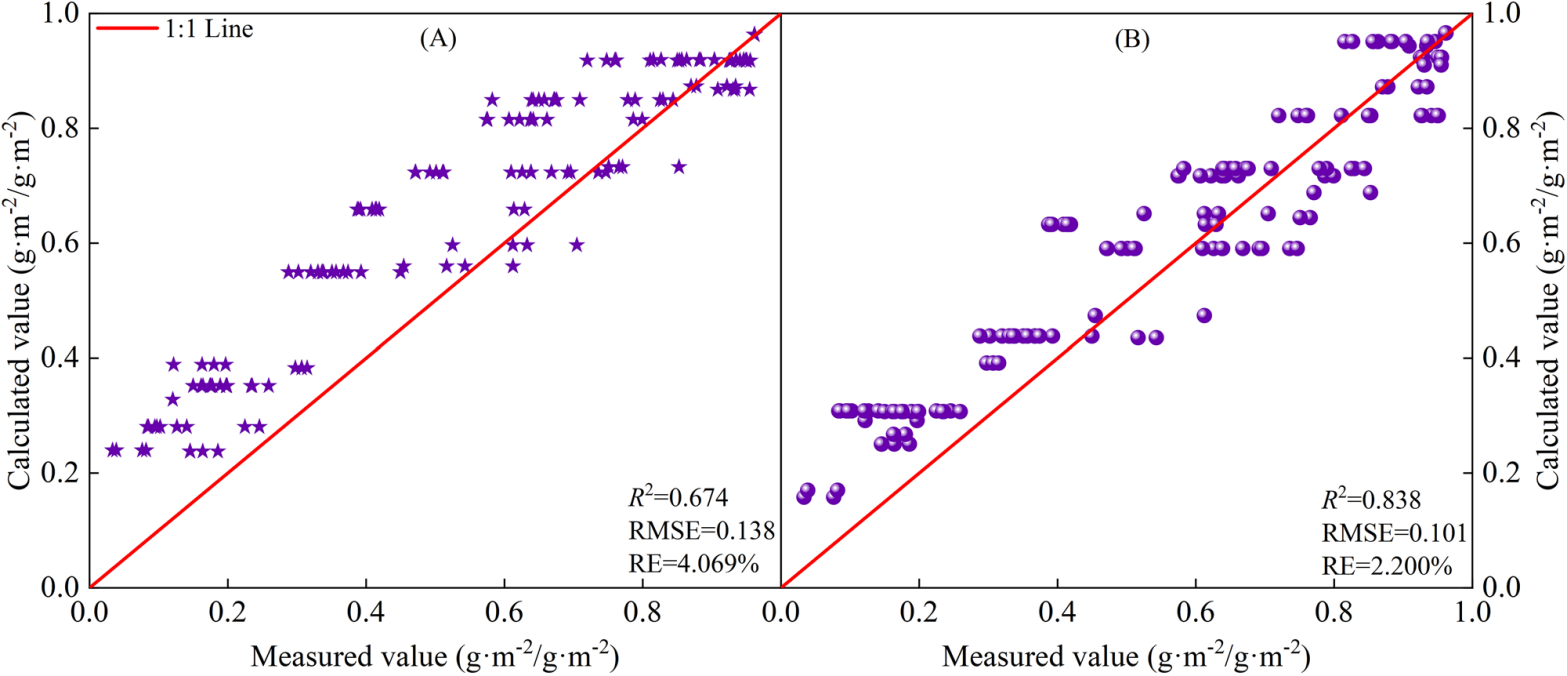
Model validation results of relative dry matter accumulation and relative cumulative growing degree days (**A**), relative cumulative reference crop evapotranspiration (**B**).

The based on *CGDD* and *CET*_*O*_ model fitting and validation results are shown in Figs. 12 and 13, with *R*^2^ ≥ 0.674, RMSE ≤ 0.138, and RE ≤ 4.069%. Similar to *H* and *LAI*, the fitting effect when using *R*_*CETo*_ as the independent variable was better than that when using *R*_*CGDD*_, and the discrete level of the measured values was smaller than that of *R*_*CGDD*_. Therefore, by using *R*_*CETo*_ as the independent variable, a cotton growth index model with more accurate fitting results can be established. Such a model reflects the cotton growth process more precisely.

### Growth model comparison

**Figure 14:**
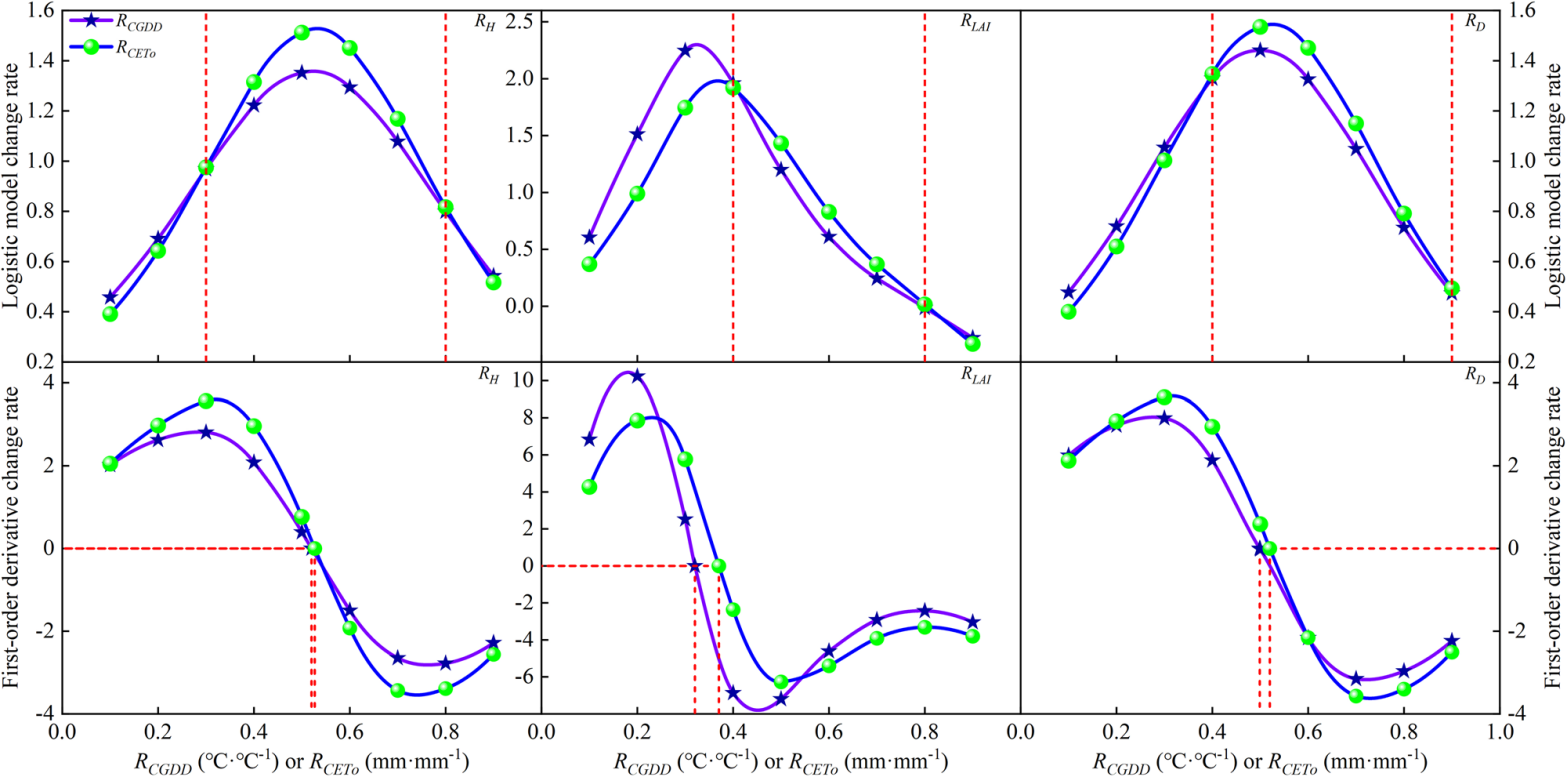
Cotton growth index logistic model and its first-order derivative change rate curve with relative cumulative growing degree days and relative cumulative reference crop evapotranspiration.

It can be seen from Fig. 14 that with the changes in *R*_*CGDD*_ and *R*_*CETo*_, the rate of change in *R*_*LAI*_ was greater than in *R*_*H*_ and *R*_*D*_ in the early stage of cotton growth. Conversely, the rate of change in *R*_*LAI*_ was smaller than in *R*_*H*_ and *R*_*D*_ in the later stage of cotton growth. This proves that suitable meteorological conditions significantly impact the form of *D* in the later stages of cotton growth, during which the energy absorbed is mainly used for the growth of the cotton bolls. This growth pattern is similar to that of other crops, such as potatoes^23^ and rice^9^. The rate of change in the *CET*_*O*_-based model was greater than in the *CGDD*-based model in the middle and later stages of cotton growth. This could be attributed to the fact that cotton growth is sensitive to various meteorological conditions, such as solar radiation, temperature, humidity, etc., which are included in the *CET*_*O*_ calculation process. This further demonstrates that *CET*_*O*_ is preferable for describing the cotton growth process.

In addition, Eqs. (13)–(18) can be used to find the second derivative and obtain the change process of the first derivative function of the logistic model with *R*_*CGDD*_ and *R*_*CETo*_. The figure shows that the change rate and inflection points based on the *CGDD* and *CET*_*O*_ logistic models were different. Therefore, although the *CGDD* calculation is simple and the *CET*_*O*_ calculation is complex, *CET*_*O*_ should be used when modeling to ensure that the model reflects the impacts of meteorological conditions on cotton growth, which *CGDD* is unable to do.

Cotton boll growth relies on the contributions from all parts of the plant, as reflected by the growth indexes of *H*, *LAI*, and *D*. To comprehensively examine the effects of meteorological conditions on cotton growth and compare with traditional methods, we analyzed the impacts of *CGDD* and *CET*_*O*_ on cotton *H*, *LAI*, and *D*. The results indicate that using *CGDD* alone to describe cotton growth yields a relatively simple relationship that does not fully reflect the impacts of meteorological conditions. Due to the spatial heterogeneity caused by meteorology conditions, cotton growth varies across regions, leading to spatial differences in yield. Therefore, it is crucial to consider meteorology conditions when describing the cotton growth process. Although differences in cotton *H*, *LAI*, and *D*, as well as irrigation water, directly affect yield, leaves are the main body for crop photosynthesis and transpiration. Therefore, further quantitative studies on the relationship between cotton *LAI* and yield, as well as irrigation water, are necessary.

### Relationship of maximum leaf area index with cotton yield and irrigation amount

**Figure 15:**
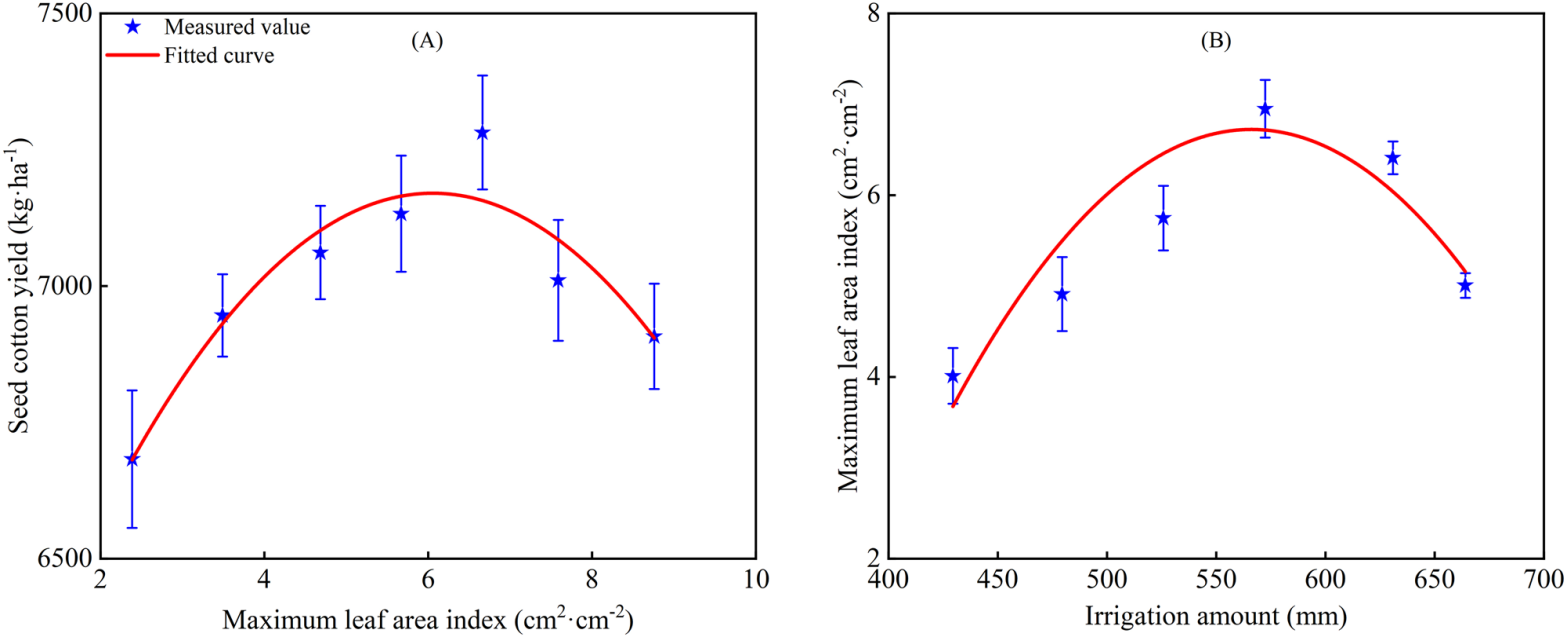
Variation trend of seed cotton yield with maximum leaf area index (**A**) and maximum leaf area index with irrigation amount (**B**).

Cotton yield is determined by multiple factors, including *LAI* and irrigation water amount (*W*). Previous studies have reported that increasing leaf area contributes little towards yield, because dark respiration rate is enhanced correspondingly after leaf expansion, which was not conducive to biomass accumulation^53^. A suitable *LAI* or an suitable increase in crop *LAI* is required to achieve higher yields. In particular, higher yields are related to the sustained photosynthetic activity of leaves. Therefore, it is important to establish the relationship of *LAI*_*max*_ with *Y* and *W* to understand cotton yields (Fig. 15). The *LAI*_*max*_ range was divided into seven categories (2–3, 3–4, 4–5, 5–6, 6–7, 7–8, and 8–9), and the *W* range was divided into six categories (400–450, 450–500, 500–550, 559–600, 600–650, and 650–700 mm). The average *LAI*_*max*_ and *W* were calculated for each category.

The relationship of *LAI*_*max*_ with *Y* and *W* can be described by the quadratic polynomial functions:

19 $$ Y = - 36.9LAI_{\max }^{2} + 446LAI_{\max } + 5824 $$

20 $$ LAI_{\max } = - 1.319 \times 10^{ - 4} W^{2} + 0.1513W - 36.95 $$

The *R*^2^ of the fitted curves of *LAI*_*max*_ with *Y* and *W* with *LAI*_*max*_ were determined to be 0.888 and 0.841, respectively. The first-order derivative of Eq. (19) was 0, while *Y* was maximized (7171.7 kg/ha) for *LAI*_*max*_ values of 6.043 cm^2^/cm^2^. Similarly, the first-order derivative of Eq. (20) was 0 with a *LAI*_*max*_ of 4.938 cm^2^/cm^2^, which corresponded to a *W* of 573.541 mm. The results suggested that if *LAI*_*max*_ values were close to 6.043 cm^2^/cm^2^, the cotton yield would be higher.

**Figure 16:**
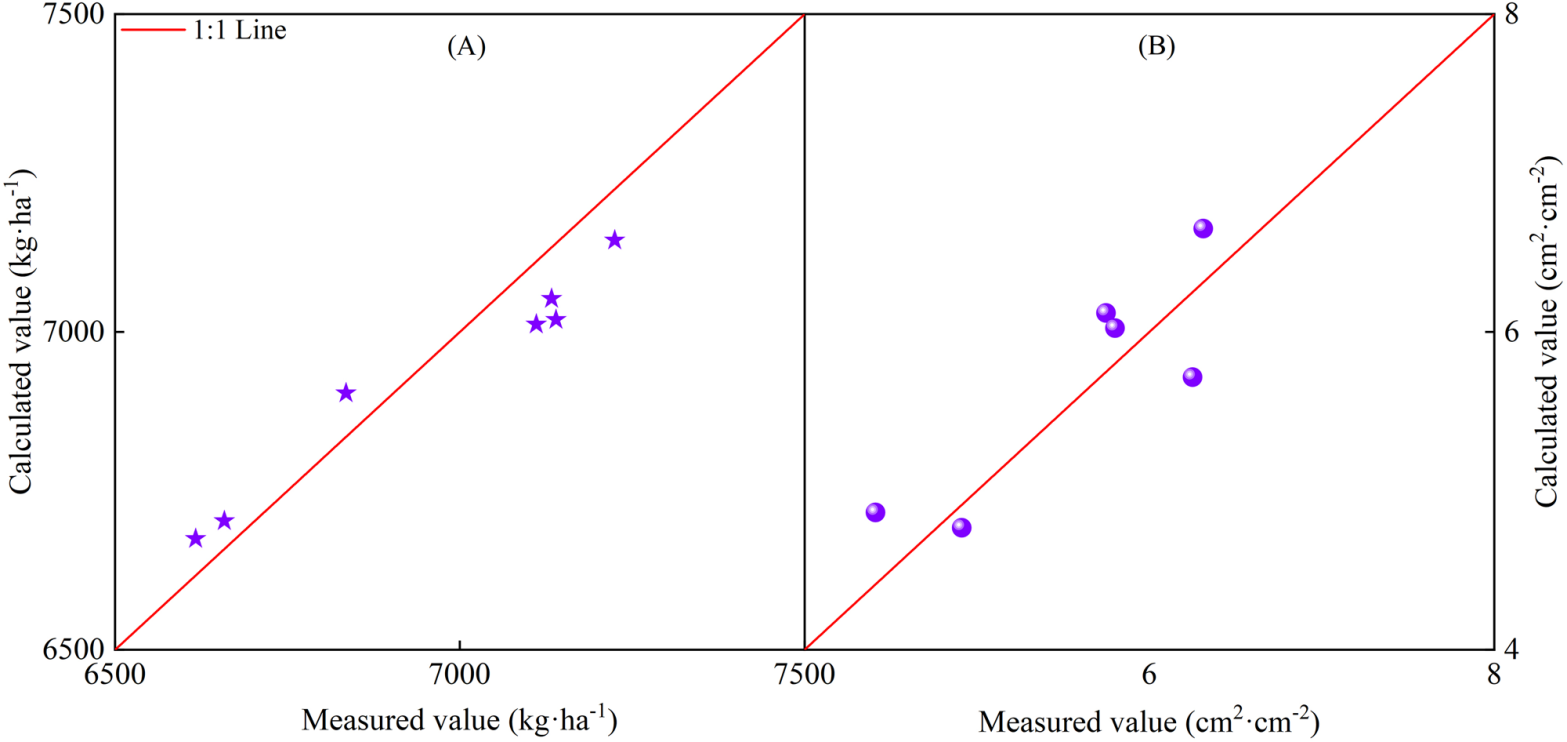
Model validation results of maximum leaf area index and seed cotton yield (**A**), irrigation amount and maximum leaf area index (**B**).

Figure 16 presents the validation results from the comparison of measured and calculated values. The results of the *Y* and *LAI*_*max*_ validations revealed *R*^2^ values of 0.791 and 0.704; RMSE values of 82.06 kg/ha and 0.404 cm^2^/cm^2^; and RE values of 0.014% and 0.431%, respectively.

Crop irrigation water utilization efficiency (*IWUE*) is a comprehensive physiological ecological index used to evaluate the degree of crop growth by examining the relationship between crop irrigation amount and yield. The *IWUE* is calculated as follows:

21 $$ IWUE = \frac{Y}{W} $$

**Figure 17:**
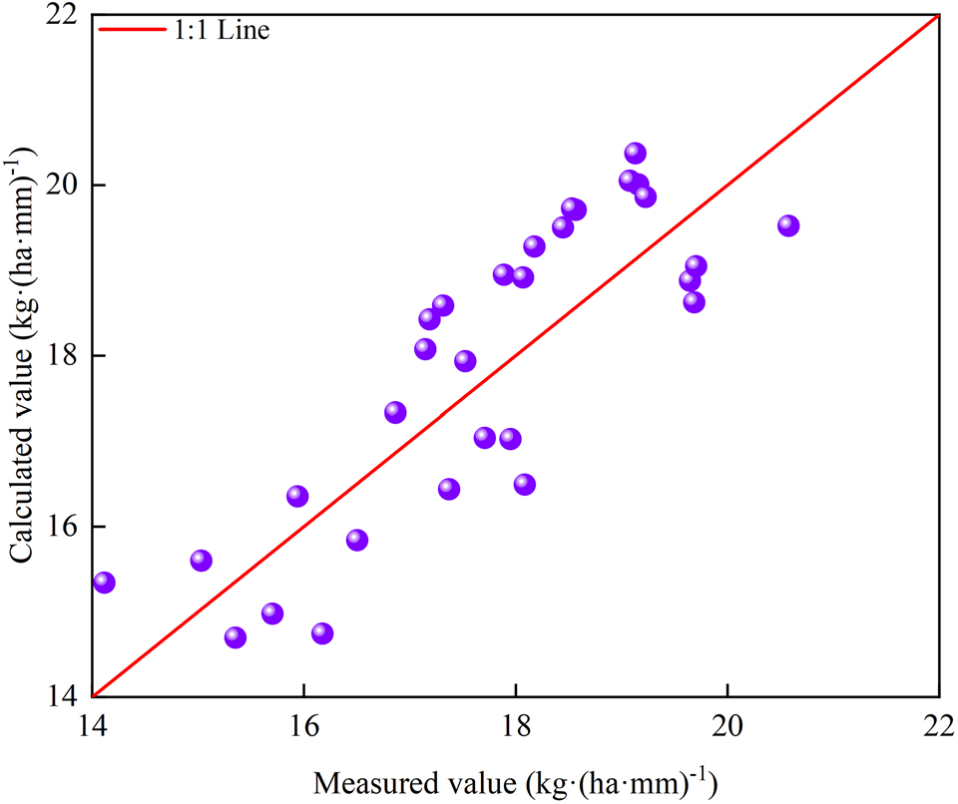
Validation results of the cotton irrigation water utilization efficiency mathematical model.

We can then obtain Eq. (22) by combining Eqs. (19)–(21). Therefore, the *IWUE* can be calculated from *W*. Figure 17 presents the validation results of the measured and calculated values. The *R*^2^, RMSE, and RE were determined to be 0.701, 0.972 kg/(ha·mm), and 0.299%, respectively.

22 $$ IWUE = \frac{{ - 6.4197 \times 10^{ - 7} W^{4} + 0.0015W^{3} - 1.2632W^{2} + 480.061W - 61035.362}}{W} $$

## Discussion

**Figure 18:**
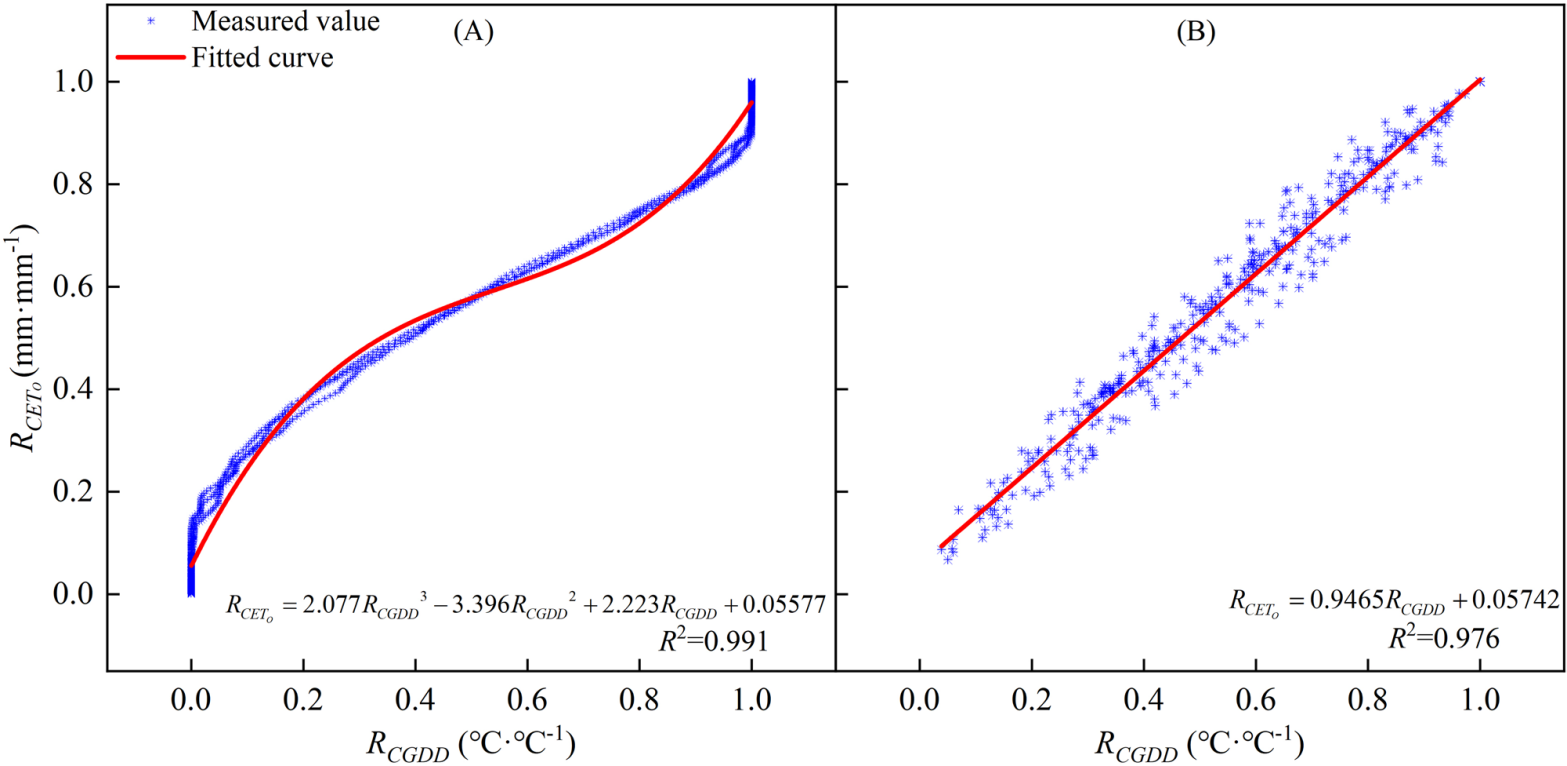
Relationships between relative cumulative growing degree days and relative cumulative reference crop evapotranspiration.

This study demonstrates the important roles that *CGDD* and *CET*_*O*_ play in simulating crop growth, and highlights the need to investigate the relationship between them. Su^28^ conducted an analysis of *CGDD* trends and the correlation between *CGDD* and *CET*_*O*_ in the Turpan area of China. Their findings indicate a strong correlation between *R*_*CGDD*_ and *R*_*CETo*_, and suggest that a cubic polynomial function (Eq. (23), Fig. 18 (A)) can accurately describe the relationship between grape budding and maturity.

23 $$ R_{{CET_{O} }} = gR_{CGDD}^{3} + hR_{CGDD}^{2} + kR_{CGDD} + l $$

The logistic growth model can be expressed as follows:

24 $$ \left\{ \begin{gathered} R_{{y_{1} }} = \frac{{y_{1} }}{{y_{1_{\max } }}} = \frac{1}{{1 + e^{{d + e(gR_{CGDD}^{3} + hR_{CGDD}^{2} + kR_{CGDD} + l)}} }} \hfill \\ R_{{y_{2} }} = \frac{{y_{2} }}{{y_{2_{\max }} }} = \frac{1}{{1 + e^{{d + e(gR_{CGDD}^{3} + hR_{CGDD}^{2} + kR_{CGDD} + l) + f(gR_{CGDD}^{3} + hR_{CGDD}^{2} + kR_{CGDD} + l)^{2} }} }} \hfill \\ \end{gathered} \right. $$

where *g*, *h*, *k*, and *l* are model fitting parameters; *y*_1_ is cotton *H* or *D*; and *y*_2_ is cotton *LAI*.

However, the cubic polynomial function has many parameters and complex forms, making it difficult to calculate when performing the equation transformation. In this study, we found that the relationship between *R*_*CGDD*_ and *R*_*CETo*_ of different regions can be approximated using a linear function (Eq. (25), Fig. 18B). The linear function has fewer parameters, so it is simpler in form and easier to calculate. The linear relationship function between *R*_*CGDD*_ and *R*_*CETo*_ uses *R*_*CGDD*_ as the independent variable as follows:

25 $$ R_{{CET_{O} }} = mR_{CGDD} + n $$

where *m* and *n* are model fitting parameters.

Combining Eqs. (6) –(8), Eqs. (10) –(12), and Eq. (25), the relationship between the model parameters *d*_1_, *d*_2_, *d*_3_, *e*_1_, *e*_2_, *e*_3_, and *f*_2_ and *a*_1_, *a*_2_, *a*_3_, *b*_1_, *b*_2_, *b*_3_, and *c*_2_ can be expressed as:

26 $$ a_{1} + b_{1} R_{CGDD} = (d_{1} + e_{1} n) + e_{1} mR_{CGDD} $$

27 $$ d_{1} = a_{1} - \frac{{b_{1} n}}{m} $$

28 $$ e_{1} = \frac{{b_{1} }}{m} $$

29 $$ a_{2} + b_{2} R_{CGDD} + c_{2} R_{CGDD}^{2} = (d_{2} + e_{2} n + f_{2} n^{2} ) + (2f_{2} mn + e_{2} m)R_{CGDD} + f_{2} m^{2} R_{CGDD}^{2} $$

30 $$ d_{2} = a_{2} - \frac{{b_{2} mn - c_{2} n^{2} }}{{m^{2} }} $$

31 $$ e_{2} = \frac{{b_{2} m - 2c_{2} n}}{{m^{2} }} $$

32 $$ f_{2} = \frac{{c_{2} }}{{m^{2} }} $$

Likewise:

33 $$ d_{3} = a_{3} - \frac{{b_{3} n}}{m} $$

34 $$ e_{3} = \frac{{b_{3} }}{m} $$

**Figure 19:**
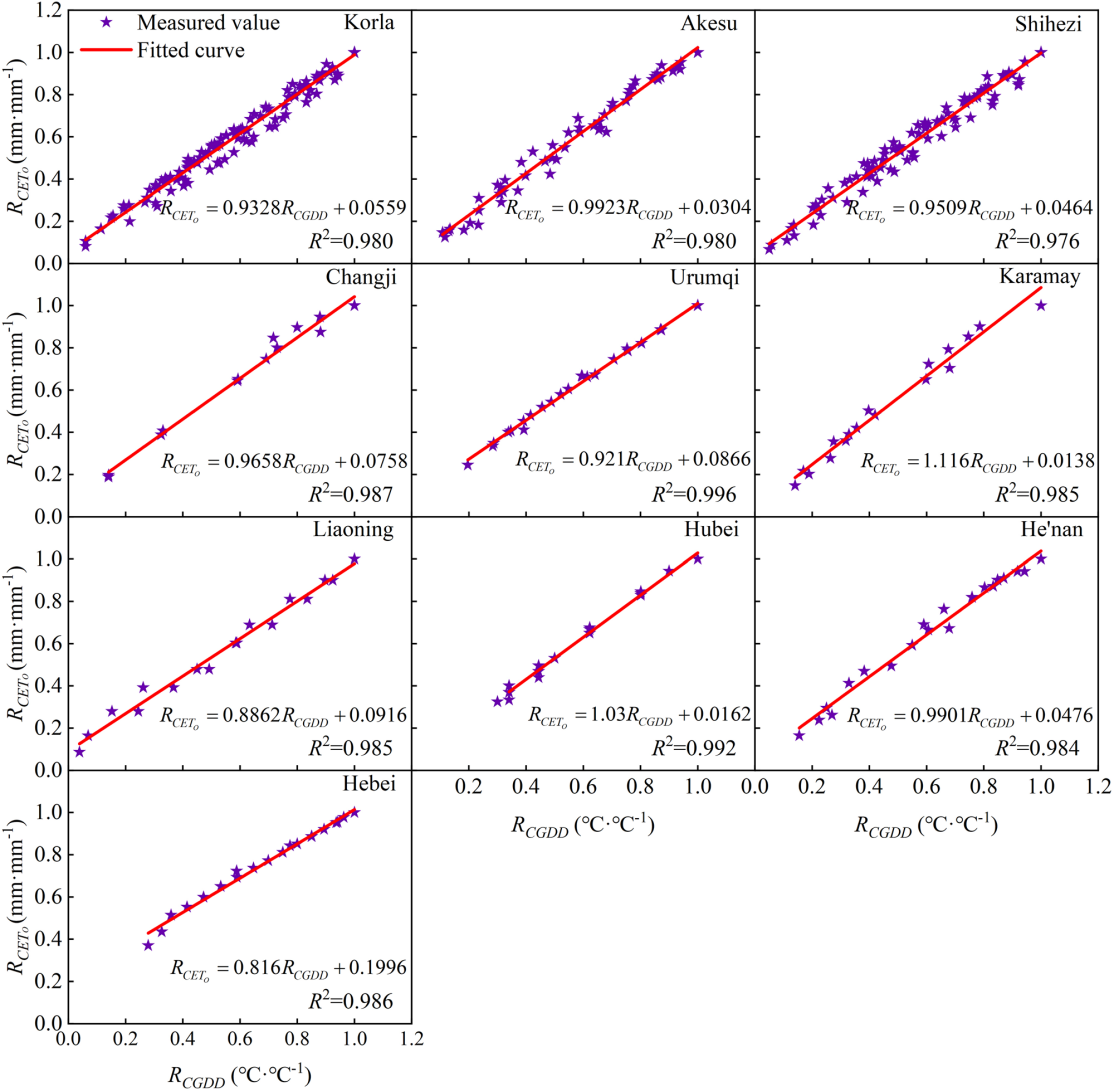
Relationships between relative cumulative growing degree days and relative cumulative reference crop evapotranspiration in every region.

We used the equations to simulate the relationship between *R*_*CGDD*_ and *R*_*CETo*_ in each region and presented the results in Fig. 19. The fitting effect had an *R*^2^ ≥ 0.976, and we obtained the values of parameters m and n for each region. Table 2 shows the validation of parameters *d*_1_, *d*_2_, *d*_3_, *e*_1_, *e*_2_, *e*_3_, and *f*_2_, which were calculated using Eqs. (26)–(34) to fit different regions. The validation of the model parameters *H* and *D* showed a good fit with RE < 0.10. However, the *LAI* model parameter validation showed unsatisfactory results. We used the modified logistic model to simulate the *LAI* change process, but this model was not well-suited for the task due to the quadratic polynomial exponential term exp, which increased the possibility of error during equation transformation. Nonetheless, it is possible to approximately calculate parameters *d*, *e*, and *f* from parameters *a*, *b*, and *c* for convenient calculations or situations where meteorological data are lacking to simulate the crop growth process.

**Table 2:** Comparison of the fitting and calculation results of parameters *d*, *e*, and *f*.

Index	Region	$a$	$b$	$c$	$d_{fit}$	$e_{fit}$	$f_{fit}$	$d_{cal}$	$e_{cal}$	$f_{cal}$	${\text{RE}}_{d}$	${\text{RE}}_{e}$	${\text{RE}}_{f}$
$R_{H}$	Korla	3.190	− 5.969	–	3.525	− 6.841	–	3.548	− 6.399	–	0.0065	0.0646	–
	Akesu	2.960	− 5.376	–	2.980	− 5.184	–	3.124	− 5.418	–	0.0485	0.0451	–
	Shihezi	3.199	− 5.425	–	3.676	− 5.296	–	3.463	− 5.705	–	0.0578	0.0773	–
	Changji	2.461	− 6.571	–	2.769	− 6.352	–	2.977	− 6.804	–	0.0751	0.0711	–
	Urumqi	3.244	− 7.648	–	4.001	− 8.201	–	3.979	− 8.304	–	0.0054	0.0126	–
	Total	2.820	− 5.424	–	3.212	− 6.091	–	3.149	− 5.731	–	0.0196	0.0592	–
$R_{LAI}$	Korla	2.266	0.899	− 10.490	1.985	2.491	− 12.430	2.174	2.312	− 11.246	0.0954	0.0718	0.0953
	Akesu	5.415	− 19.380	12.490	5.502	− 19.075	12.629	6.020	− 20.301	12.587	0.0941	0.0643	0.0033
	Shihezi	2.599	− 5.198	− 2.850	2.545	− 5.928	− 2.685	2.846	− 5.174	− 2.997	0.1181	0.1272	0.1163
	Changji	2.274	5.934	− 38.620	1.798	10.997	− 35.790	1.570	12.424	− 39.988	0.1269	0.1298	0.1173
	Karamay	2.844	− 10.850	4.294	3.122	− 10.480	4.320	2.979	− 9.817	3.848	0.0460	0.0632	0.1093
	Liaoning	4.018	− 13.660	6.960	5.414	− 18.220	9.050	5.505	− 17.038	7.854	0.0167	0.0649	0.1322
	Hubei	2.462	− 8.495	6.180	2.281	− 7.467	5.350	2.597	− 8.436	6.000	0.1381	0.1298	0.1215
	He’nan	5.490	− 20.690	13.830	5.839	− 19.850	12.361	6.517	− 22.240	13.968	0.1161	0.1204	0.1300
	Hebei	7.650	− 28.670	20.850	13.511	− 40.650	21.870	15.910	− 47.635	25.551	0.1776	0.1718	0.1683
	Total	4.553	− 16.03	10.09	5.024	− 15.66	9.500	5.563	− 18.230	11.263	0.1072	0.1641	0.1856
$R_{D}$	Korla	4.452	− 7.963	–	5.319	− 9.103	–	4.930	− 8.537	–	0.0732	0.0622	–
	Akesu	2.673	− 5.846	–	2.983	− 5.655	–	2.852	− 5.891	–	0.0440	0.0418	–
	Shihezi	2.557	− 5.557	–	2.990	− 6.246	–	2.828	− 5.844	–	0.0542	0.0644	–
	Liaoning	2.352	− 6.638	–	3.250	− 8.025	–	3.038	− 7.490	–	0.0651	0.0666	–
	Hubei	4.693	− 9.715	–	4.771	− 9.564	–	4.846	− 9.432	–	0.0157	0.0138	–
	He’nan	5.942	− 10.400	−	5.978	− 9.726	−	6.442	− 10.504	−	0.0776	0.0800	−
	Total	2.877	− 5.764	−	3.206	− 6.163	−	3.227	− 6.090	−	0.0065	0.0119	−

Note: *d*_*fit*_, *e*_*fit*_, and *f*_*fit*_ were fitted, *d*_*cal*_, *e*_*cal*_, and *f*_*cal*_ were calculated by Eqs. (26)–(34).

Climate suitability is an important ecological characteristic for crops and forms the basis of the distribution of crop production^54^. Cotton is sensitive to climate variability and is unable to resist damage caused by drought and cold^55^. Numerous studies have reported quantitative predictions of the impact of future climate change on cotton production^56–60^. These studies generally predict major changes in the trends of cotton production under future climate scenarios, and describe how cotton agricultural production can respond and adapt to climate change. However, discrepancies among research methods (such as crop models, climate models, and climate scenarios) create uncertainties regarding the extent and severity of the impacts that future climate change will have on cotton yields. Therefore, future research should focus on quantitatively exploring the relationship between climate change and cotton growth process.

Many mathematical models have been used to describe crop growth processes, such as the Logistic, Richards, Gompertz, Hoerl, and others. However, previous research has shown that the Logistic model has a better fitting effect when using time or *CGDD* as independent variables^11,12^. While many studies have quantitatively analyzed the impact of *CGDD* on crop growth, including for winter wheat^8^, rice^9^, cotton^24^, potato^23^, maize^61^, and watermelon^26^. It is important to note that these models have some limitations, as *CGDD* considers only a few meteorological factors, such as the highest daily temperature and the lowest daily temperature. However, meteorology factors such as temperature, wind, rainfall, relative humidity, and sunshine duration significantly affect the production of cotton flowers and bolls^20^. For instance, temperature is the most critical meteorology factor that affects cotton yield, and heat stress can lead to reduced fruit retention, delayed crop maturity, and lower lint quality. Strong winds may also cause boll shedding, thereby reducing yield. Additionally, continuous rainfall during flowering and boll opening can impair pollination and potentially reduce fiber quality^31^. Moreover, climate change is a crucial issue worldwide, and its negative effects on cotton growth and development can result from an increased number and severity of days with very high temperatures during the cotton season. These events can lower cotton yields by decreasing daily photosynthesis and, at times, raising respiration at night^20,31,56,57^. Further research is necessary to comprehensively consider these factors when simulating cotton growth. In this study, we present a new cotton growth model that try to overcomes some of these limitations and demonstrate its superiority over existing models through comparison and analysis. Specifically, we use the *CET*_*O*_, which comprehensively considers meteorology conditions such as local solar radiation, humidity, temperature, and wind speed. Our results showed that the *CET*_*O*_-based logistic model more accurately described the cotton growth process compared with the *CGDD*-based model. We deeply evaluated both *CET*_*O*_ and *CGDD* as effective predictors when simulating crop growth processes. In future studies, we plan to make further improvements to this method and expand our research to other crops. These research results and the constructed mathematical models can provide a scientific basis for improving agricultural production efficiency while also contributing to the improvement and development of crop growth models.

## Conclusion

To provide a more accurate description of the effects of climate change on cotton growth, this paper proposes a novel logistic cotton growth model that uses *CET*_*O*_ instead of *CGDD* as the independent variable. Although crop growth and yields varied significantly across different regions and years, the overall trends in their developments throughout the year remained consistent. A comparison between models using *CET*_*O*_ and *CGDD* as independent variables tested the precision of the former in simulating crop growth. The results showed that *CET*_*O*_ can comprehensively reflect the impacts of meteorological factors on cotton growth and more accurately describe the cotton growth process. Additionally, this study established mathematical models relating cotton *LAI*_*max*_ to *Y* and *W*. When cotton *Y* reached its maximum value of 7171.7 kg/ha, the value of *LAI*_*max*_ was 6.043 cm^2^/cm^2^. The required *W* was 518.793 mm, and the *IWUE* was 21.153 kg/(ha·mm). In the future, more attention should be paid to the impacts of meteorological conditions and irrigation on crop growth.

## Data Availability

The datasets generated and/or analysed during the current study are available from the corresponding author on reasonable request.
